# Functional tooth unit, periodontal status, and association with weight change in older adults

**DOI:** 10.1002/jper.70100

**Published:** 2026-03-06

**Authors:** Mariana S. Muñoz, Natália M. Pola, Juliana B. Hilgert, Fernando N. Hugo, Roberto P. Pimentel, Eleanor M. Simonsick, Francisco W. M. G. Muniz

**Affiliations:** ^1^ Graduation Program in Dentistry Federal University of Pelotas Pelotas Brazil; ^2^ Semiology and Clinic Department Federal University of Pelotas Pelotas Brazil; ^3^ Department of Preventive and Social Dentistry Federal University of Rio Grande do Sul Porto Alegre Brazil; ^4^ Department of Epidemiology and Health Promotion New York University College of Dentistry New York USA; ^5^ Longitudinal Studies Section National Institute on Aging Intramural Research Program Baltimore Maryland USA

**Keywords:** aged, body weight changes, tooth loss, weight gain

## Abstract

**Background:**

Literature reports that having fewer teeth is associated with reduced chewing capacity, which in turn may lead to weight changes due to diminished oral functionality. This study aims to assess the association between functional tooth units (FTU) and periodontal status and weight change among older adults participating in the Health, Aging, and Body Composition Study (Health ABC).

**Methods:**

This study followed individuals with complete data on weight at years 2 and 6 of Health ABC. Variables included the number of teeth, FTU, and periodontal status, which were considered the primary exposures. The main outcome was a weight change of at least 5% during the follow‐up period. Multinomial regression was used to calculate the odds ratio (OR) associated with at least 5% weight change. Independent adjusted models were built for each primary exposure (α < 5%).

**Results:**

The study included 903 participants, of whom 231 (25.6%) experienced weight loss and 104 (11.5%) experienced weight gain. No significant association was found between weight loss and any of the exposures (*p* > 0.05). However, weight gain was associated with clinical attachment loss (CAL), number of teeth (OR: 0.97; 95%CI: 0.94–0.99), FTU/molars (OR: 0.83; 95%CI: 0.70–0.98), FTU/posterior (OR: 0.92; 95%CI: 0.84–0.99), and FTU/total (OR: 0.95; 95%CI: 0.91–0.99).

**Conclusion:**

Tooth loss was associated with weight gain over 4 years. Additional research is needed to uncover underlying mechanisms, including food choice.

**Plain language summary:**

Researchers studied how tooth loss and gum health affect weight changes in older adults. They followed participants from the Health, Aging, and Body Composition Study for 4 years, looking at their teeth, chewing ability, and weight changes.

Out of 903 people in the study, 231 (25.6%) lost weight, and 104 (11.5%) gained weight. The results showed that having fewer teeth and poor gum health did not significantly affect weight loss. However, weight gain was linked to tooth loss and gum problems. People with fewer teeth or weaker chewing ability were more likely to gain weight.

The study suggests that losing teeth may lead to changes in eating habits that result in weight gain. However, more research is needed to understand how tooth loss affects food choices and overall health.

## 1 INTRODUCTION

Weight change is inevitable over the life course, but for older adults, weight change has been associated with age‐related adverse health outcomes.^1^ Unintentional and significant weight loss among older adults can lead to sarcopenia and low bone density.^2^ In contrast, obesity is a chronic condition characterized by excess body fat, causing damage to the individual's health.^3^ Both malnutrition and obesity are important public health problems resulting from complex relationships between genetic, socioeconomic, and cultural influences.^4^, ^5^ Urban development, lifestyle habits, and food consumption patterns have been associated with obesity, which has increased by 27.5% in the past 3 decades globally.^4^

The literature shows that obesity is associated with low masticatory capacity.^6^ A higher number of missing teeth, combined with the absence of prosthetic rehabilitation, negatively affects individuals' abilities to eat.^7^ Furthermore, with advancing age, oral diseases such as periodontitis, the leading cause of tooth loss, become more common. These diseases, in turn, can influence body weight differently. Tooth mobility resulting from periodontitis progression and periodontal tissue breakdown leads to reduced chewing capacity, which in turn causes individuals to avoid nutrient‐rich, harder‐to‐chew foods and prefer softer, caloric‐dense foods,^8^ resulting in weight gain. Conversely, severe periodontal tissue breakdown and the consequent impairment in masticatory efficiency, along with increased systemic inflammation characteristic of periodontal inflammation, can lead to a reduction in appetite,^9^ potentially resulting in unintentional weight loss. Evidence from one study^10^ further supports that the presence and extent of periodontal disease‐related parameters are associated with an increased risk of weight loss among older adults.

In this context, the absence or reduction in the number of functional dental units (FDUs), described as pairs of opposing teeth,^11^ impairs chewing. The subsequent mechanical reduction of food into smaller pieces affects swallowing and digestion, which in turn directly compromises nutritional intake.^12^ As a result, dietary changes may occur in the presence of tooth loss, with the replacement of hard‐to‐chew foods with softer ones. Therefore, these alterations may significantly contribute both weight gain and weight loss among these individuals.^12^, ^13^

Softer foods, which are generally more processed, tend to be higher in fat, cholesterol^14^, and sugars, making them more calorically dense. Some individuals may experience a progressive increase in weight with advancing age, which can result in obesity,^15^ as a result of a more sedentary lifestyle. In contrast, in other individuals, the reduction in FTUs can induce inadequate food selection, leading to an adaptation of food textures according to dental status and a decrease in appetite. This can lead to swallowing disorders that result in weight loss and even malnutrition.^16^ Both weight loss and gain are associated with an increased risk of all‐cause mortality in older adults.^17^

In this context, although previous studies have primarily emphasized tooth loss as a determinant of weight gain, the present study not only aligns with this viewpoint but also advances understanding by employing a prospective cohort design. Specifically, it examines the associations between FTUs and periodontal status with both weight gain and weight loss among older adults enrolled in the Health, Aging, and Body Composition Study (Health ABC). The hypothesis was that having fewer FTUs and poorer periodontal health at baseline would lead to significant weight changes during the 4‐year follow‐up period.

## 2 MATERIALS AND METHODS

### 2.1 Study design

This is a prospective cohort study, initiated in 1997–1998 in Pittsburgh, Pennsylvania, and Memphis, Tennessee, in the United States. All participants gave written informed consent, and the institutional review boards at both sites approved all study protocols (03AG0325 / CR000456). The present study is a secondary analysis of Health ABC, an interdisciplinary study aiming to investigate whether changes in body composition, weight‐related health conditions, and behavioral factors contribute to functional decline and loss of independence in older adults. All study procedures were carried out following the declaration of Helsinki of 1975, and this report follows the guidelines of the Strengthening the Reporting of Observational Studies in Epidemiology checklist.^18^

### 2.2 Study population, inclusion and exclusion criteria

Health ABC randomly selected 3,075 participants, residing within a 2‐hour drive of the clinical centers in Memphis and Pittsburgh. They were contacted by mail, followed by a telephone interview to assess eligibility. To be included, participants had to declare no difficulty walking 1‐quarter of a mile, climbing ten steps without resting, or performing basic daily activities, had no life‐threatening illness, had not had cancer treatment in the previous 3 years, were aged 70–79, and had no intention of moving out of the study region in the next 3 years. They were assessed at the beginning of the study, annually in person in years 2 through 6, and then biannually in years 8 and 10, with interim telephone follow‐ups every 6 months.

In this study, individuals with complete data on weight measures at year 2 (oral health sub‐study, baseline of this report) and year 6, along with oral health examination information that comprised the number of teeth, FTUs, and periodontal health (probing depth [PD] or clinical attachment level [CAL]), were included.

### 2.3 Oral health assessment

The oral health assessment was performed once (during year 2 or 3) and included a full‐mouth periodontal examination and tooth count. Periodontal measurements were obtained by a trained periodontist, while the tooth count and soft and hard tissue assessments were performed by calibrated dental hygienists, each assisted by a data recorder. All examiners completed an extensive training and calibration program before fieldwork, using a senior periodontist as the gold standard. A minimum of 90% agreement in PD and CAL was required and achieved before data collection. Two additional calibration checks were conducted during the study to ensure consistency across both study sites (Memphis and Pittsburgh).^10^ Periodontal probing was performed using a UNC‐15 probe (Hu‐Friedy, Chicago, IL), with measurements recorded at 6 sites (mesiobuccal, buccal, distobuccal, mesiolingual, lingual, and distolingual) on all present teeth. PD was measured from the gingival margin to the base of the pocket, and CAL was calculated as the distance from the cemento‐enamel junction (CEJ) to the gingival margin, subtracting when the gingival margin was coronal to the CEJ and adding when apical.

### 2.4 Main outcome, exposures, and covariates

The main study outcome was a change of at least 5% in weight over 3 to 4 years.^19^ This parameter was used considering that this threshold of weight loss is predictive of mortality in older adults,^20^ all‐age adults,^21^ and in patients with heart failure.^22^ In the present study, participants' weight was measured in kilograms every year between years 2 and 6 using a standard balance scale. Therefore, participants were categorized into 3 distinct groups: *stable*, those whose weight did not change by 5% or more in either direction over the study period; *weight loss*, those who experienced a reduction of at least 5%; and *weight gain*, those who experienced an increase of at least 5%.

**FIGURE 1:**
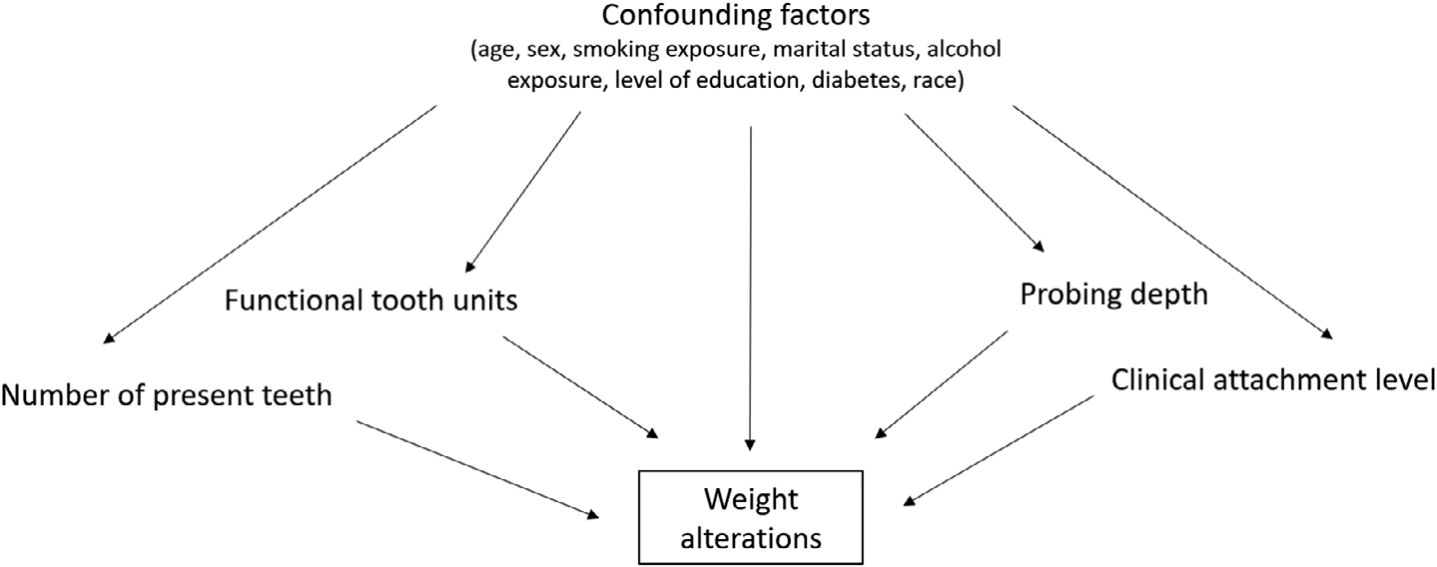
DAG for the possible association between weight alterations among older adults and exposure variables. DAG, directed acyclic graph.

The selection of covariables for this study was based on a previous literature review identifying the most important factors that might influence weight changes among older adults, as well as the availability of the covariables in the Health ABC dataset. Sociodemographic variables included sex (male or female), age (in years), race (white or black), marital status (single, married, widowed, or divorced), and schooling (up to 8^th^ grade, grade 9^th^ to 12^th^, or higher education). Behavioral variables included smoking (never, former, or current) and alcohol exposure (none/less than 1 drink per week or at least 1 drink per week). The medical variable analyzed was diabetes (yes or no), in which participants answered the following question: “Has a doctor ever told you that you have diabetes?” Participants who were receiving drug treatments for diabetes were also categorized as “yes.” For the abovementioned variables, the answers provided in the baseline appointment were considered. Use of dental prosthesis and self‐reported appetite and eating behaviors were also considered as potential covariates. However, due to the high number of missing data among the included participants, these variables were not included in the analysis. Figure 1 presents the directed acyclic graph (DAG) illustrating the possible associations between the outcome and all independent variables, including the following primary exposures.

Variables related to periodontal examination were included in the analysis, such as the number of present teeth, the number of individuals with at least 2 sites with PD ≥5 mm (yes or no), mean PD (in mm), and mean CAL (in mm). PD clinical parameters were applied to include individuals diagnosed with at least stage II periodontitis.^23^

FTUs were defined as pairs of opposing teeth in occlusion, excluding third molars. Teeth restored with any fixed prostheses or crowns were also considered functional and scored equivalently to natural teeth. FTUs were categorized by region: molar, premolar, anterior, posterior (premolars + molars), and total (all regions). Each category was based on specific occlusal pairs:

- Molar FTU: 17–47, 16–46, 27–37, and 26–36
- Premolar FTU: 14–44, 15–45, 24–34, and 25–35
- Anterior FTU: 11–41, 12–42, 13–43, 21–31, 22–32, and 23–33
- Posterior FTU: All molar and premolar pairs listed above
- Total FTU: All anterior and posterior pairs combined

Additionally, FTU scores were calculated to reflect chewing potential. Molar pairs were scored as 2 units, while premolar and anterior pairs were scored as 1 unit each. Two score variables were defined:

- Posterior FTU Score: Sum of occluding premolar and molar pairs (natural or restored), ranging from 0 to 12;
- Total FTU Score: Sum of all occluding pairs (natural or restored), ranging from 0 to 18.

Both periodontal status and FTU categories were defined as exposures.

### 2.5 Statistical analysis

Statistical analyses were conducted employing SPSS, version 29.0 (SPSS, version 29.0, IBM Corp., Armonk, NY, USA). Bivariate analysis was performed using 1‐way analysis of variance (ANOVA), with a Tukey's *post‐hoc* test when a symmetrical data distribution was detected. The Kruskal–Wallis test was utilized to examine asymmetrical continuous variables. Bivariable and multivariable analyses were specifically conducted for the weight loss and weight gain outcomes (no weight alteration was considered the reference group), utilizing multinomial regressions to estimate the odds ratios (OR) and their 95% confidence intervals (95% CIs).

All independent variables included in the DAG were deemed important and, thus, included in the final adjusted models. No high correlation coefficients were identified between any pair of independent variables (whenever statistically significant, Pearson correlation coefficients ranged between 0.3 and −0.3). Overall, these coefficients support the stability of the multivariable modelling used.

The adequacy of the multinomial logistic regression model was assessed using the Pearson chi‐square goodness‐of‐fit test, which indicated that all tested models provided an acceptable fit to the data (*p* > 0.05). Overdispersion was not detected, as all dispersion parameters ranged between 1.01 and 1.04, considering all adjusted models. Moreover, multicollinearity was tested among each adjusted model, but none was detected, using the cutoff points of < 10 and 0.1 for the variance inflation factor and tolerance, respectively.

Independent adjusted models were built for each primary exposure (mean PD, mean CAL, at least 2 sites with PD ≥5 mm, number of present teeth, FTU molar, FTU premolar, FTU anterior, FTU posterior, FTU total, posterior FTU score, total FTU score). The level of significance adopted was 5%.

An artificial intelligence tool (ChatGPT, OpenAI) was used exclusively for English language editing. No scientific content, data analysis, or interpretation of results was generated by AI, and the tool is not listed as an author.

## 3 RESULTS

**FIGURE 2:**
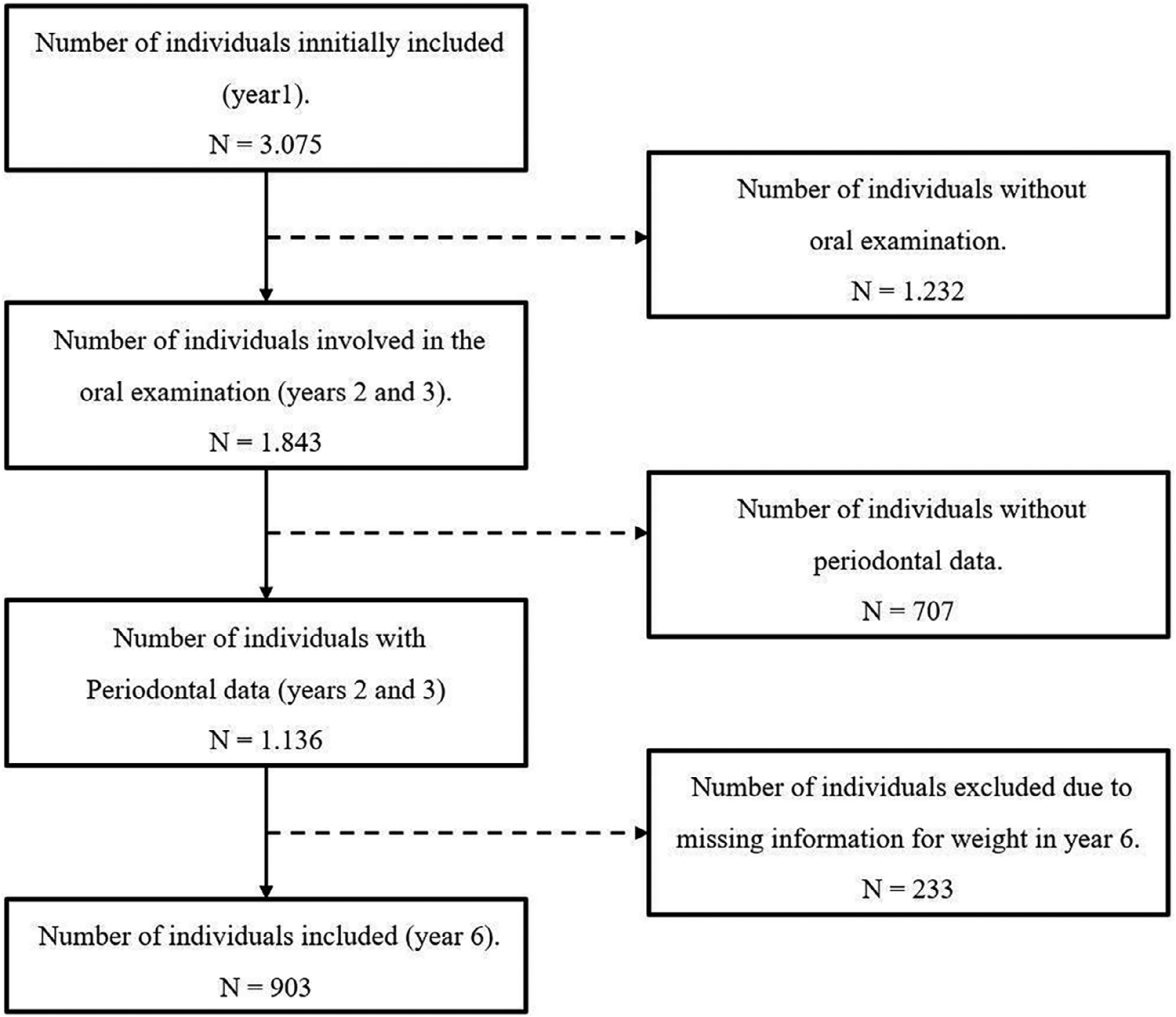
Flowchart of study participants selection and exclusion.

Out of the initial 3,075 participants enrolled in the Health ABC study in year 1, 1,843 underwent an oral examination, as part of a sub‐study, in years 2 and 3. Only participants who were assessed on days when the periodontist was present underwent a periodontal examination. This led to a total of 1,136 participants with available periodontal data. However, from this pool, 233 participants were excluded because they did not have weight data in year 6 (retention rate: 79.49%). Many of these participants were deceased or otherwise incapacitated. Figure 2 provides a flowchart of study participants' selection and exclusion.

Table S1 provides a comparison between older adults included in the present study with those without weight data in year 6. No statistically significant difference between these individuals was detected, except for marital status. A higher proportion of married participants was identified in the group of individuals included.

Table 1 shows that a total of 903 participants were evaluated and classified according to weight alterations between year 2 and year 6: no weight change (*n* = 568; 62.9%), weight loss ≥5% (*n* = 231; 25.6%), and weight gain ≥5% (*n* = 104; 11.5%). The mean percentage of weight change differed significantly among groups (*p*  <  0.001), with the weight loss group showing a mean reduction of −9.15% (SD  =  4.39), and the weight gain group showing a mean increase of 8.28% (SD  =  2.89). No statistically significant differences were observed among groups regarding sex distribution, age, race, marital status, educational level, smoking status, or diabetes prevalence (*p*  >  0.05). However, alcohol consumption at baseline was significantly different across groups (*p*  =  0.009), with the weight loss group reporting a lower frequency of alcohol intake. Dental parameters (including number of present teeth, PD, CAL, and FTU) did not differ significantly among weight change categories.

**TABLE 1:** Demographic, medical, dental characteristics of participants, classified according to weight alterations from year 2 to year 6 (*n* = 903)

Parameter	No weight alterations (*n* = 568; 62.9%)	Weight loss (≥5%) (*n* = 231; 25.6%)	Weight gain (≥5%) (*n* = 104; 11.5%)	*p*‐value
Weight alteration (in %)	−0.35 ± 2.65	−9.15 ± 4.39	8.28 ± 2.89	**<0.001#**
*Mean ± SD (median*–*IQ)*	(−0.43; −2.47–1.79)	(−7.61; −10.48 to −6.10)	(7.50; 6.02 – 9.76)	**<0.001&**
Sex				
*Male*–*n (%)*	292 (51.4)	120 (51.9)	51 (49.0)	0.881*
*Female*–*n (%)*	276 (48.6)	111 (48.1)	53 (51.0)	
Age (year 6)	78.53 ± 2.76	78.31 ± 2.78	78.57 ± 2.75	0.548$
*Mean ± SD (median*–*IQ)*	(78.00; 76.00–81.00)	(78.00; 76.00–81.00)	(78.00; 76.00–81.00)	
Race				0.112*
*White*–*n (%)*	383 (67.4)	141 (61.0)	62 (59.6)	
*Non‐White*–*n (%)*	185 (32.6)	90 (39.0)	42 (40.4)	
Marital status				0.186*
*Single*–*n (%)*	33 (6.3)	11 (5.2)	5 (5.1)	
*Married*–*n (%)*	319 (60.8)	127 (59.6)	48 (49.0)	
*Widow*–*n (%)*	133 (25.3)	56 (26.3)	30 (30.6)	
*Divorced*–*n (%)*	40 (7.6)	19 (8.9)	15 (15.3)	
*Missing*	43	18	6	
Schooling				0.253*
*Up to 8^th^ Grade*–*n (%)*	32 (5.6)	18 (7.9)	7 (6.7)	
*Grade 9^th^ to 12^th^*–*n (%)*	209 (36.9)	90 (39.5)	30 (28.8)	
*Higher*–*n (%)*	326 (57.5)	120 (52.6)	67 (64.4)	
*Missing*	1	3	0	
Smoking exposure at 1^st^ Year				0.784*
Never‐n (%)	270 (47.6)	102 (44.3)	47 (45.2)	
*Current*–*n (%)*	43 (7.6)	23 (10.0)	8 (7.7)	
*Former*–*n (%)*	254 (44.8)	105 (45.7)	49 (47.1)	
*Missing*	1	1	0	
Alcohol exposure at 1^st^ year				0.009*
*None or < 1 per week*–*n (%)*	364 (64.2)	172 (75.4)	70 (67.3)	
*At least 1 per week*–*n (%)*	203 (35.8)	56 (24.6)	34 (32.7)	
*Missing*	1	3	0	
Diabetes at 1^st^ year				0.558*
*No*–*n (%)*	502 (88.4)	203 (87.9)	88 (84.6)	
*Yes*–*n (%)*	66 (11.6)	28 (12.1)	16 (15.4)	
*Missing*	0	0	0	
No. of teeth present	19.86 ± 7.52	19.61 ± 7.79	18.17 ± 8.07	0.153$
*Mean ± SD (median*–*IQ)*	(22.00; 15.00–26.00)	(21.00; 13.00–26.00)	(20.00; 11.00–25.00)	
PD	2.04 ± 0.76	2.10 ± 0.87	2.12 ± 0.76	0.647$
*Mean ± SD (median*–*IQ)*	(1.93; 1.54–2.44)	(1.96; 1.54–2.45)	(1.95; 1.64–2.54)	
At least 2 sites with PD ≥5 mm				0.557*
*No*–*n (%)*	311 (54.8)	117 (50.6)	57 (54.8)	
*Yes*–*n (%)*	257 (45.2)	114 (49.4)	47 (45.2)	
CAL	2.28 ± 1.42	2.30 ± 1.37	2.66 ± 1.85	0.186$
*Mean ± SD (median*–*IQ)*	(1.85; 1.32–2.84)	(1.91; 1.37–2.98)	(2.21; 1.42–3.17)	
*Missing*	1	0	0	
FTU Molar	1.39 ± 1.52	1.38 ± 1.58	1.08 ± 1.36	0.143$
*Mean ± SD (median*–*IQ)*	(1.00; 0.00–3.00)	(0.00; 0.00–3.00)	(0.00; 0.00–2.00)	
FTU Anterior	4.30 ± 2.28	4.08 ± 2.41	3.88 ± 2.44	0.167$
*Mean ± SD (median*–*IQ)*	(5.00; 3.00–6.00)	(6.00; 2.00–6.00)	(5.00; 1.25–6.00)	
FTU Premolar	2.06 ± 1.58	2.00 ± 1.63	1.78 ± 1.71	0.257$
*Mean ± SD (median*–*IQ)*	(2.00; 0.00–4.00)	(2.00; 0.00–4.00)	(2.00; 0.00–3.75)	
FTU posterior	3.45 ± 2.85	3.38 ± 2.96	2.86 ± 2.81	0.106$
*Mean ± SD (median–IQ)*	(3.00; 0.00 – 6.00)	(3.00; 0.00–6.00)	(3.00; 0.00–5.75)	
FTU total	7.75 ± 4.63	7.46 ± 4.79	6.74 ± 4.78	0.131$
*Mean ± SD (median–IQ)*	(8.00; 5.00–12.00)	(8.00; 4.00–12.00)	(7.00; 2.00–11.00)	
Posterior FTU score	4.84 ± 4.28	4.76 ± 4.45	3.93 ± 4.08	0.083$
*Mean ± SD (median–IQ)*	(4.00; 0.00–8.00)	(4.00; 0.00–9.00)	(3.00; 0.00–8.00)	
Total FTU Score	9.14 ± 5.93	8.84 ± 6.14	7.82 ± 5.93	0.100$
*Mean ± SD (median–IQ)*	(9.00; 5.00–4.00)	(9.00; 4.00–14.00)	(7.50; 2.00–13.00)	

*Note*: Posterior FTU score: represented by FTUs posterior to canines (except third molars); Total FTU score: represented by all teeth in occlusion (except third molars). In both variables, pairs of molar teeth scored 2 units and pairs of anterior teeth and premolars scored 1 unit each.

Abbreviations: ANOVA, analysis of variance; CAL, clinical attachment level; FTU, functional tooth unit; IQ, interquartile range; PD, probing depth; SD, standard deviation.

*t‐test for independent samples.

#1‐way ANOVA.

&post‐hoc of Tukey (all pair‐wise comparison had the same *p*‐value).

$Kruskal—Wallis.

In the bivariable analysis for weight alteration (Table 2), mean CAL (OR: 1.17; 95%CI: 1.03–1.33), number of teeth (OR: 0.97; 95%CI: 0.95–0.99), total FTU (OR: 0.96; 95%CI: 0.92–0.99), posterior FTU score (OR: 0.95; 95%CI: 0.90–0.99), and total FTU score (OR: 0.96; 95%CI: 0.93–0.99) were associated with weight gain over the follow‐up period.

**TABLE 2:** Bivariable analyses for weight alteration (multinominal regression analyses)

Parameter	Weight loss OR (95% CI)	*p*‐value	Weight gain OR (95% CI)	*p*‐value
Sex				
*Male*	1.02 (0.75–1.39)	0.890	0.91 (0.60–1.38)	0.910
*Female*	Ref.		Ref.	
Age	0.97 (0.92–1.03)	0.972	1.01 (0.93–1.08)	0.895
Race				
*White*	0.76 (0.55–1.04)	0.085	0.71 (0.46–1.10)	0.122
*Non‐White*	Ref.		Ref.	
Marital status				
*Single*	0.70 (0.29–1.68)	0.427	0.40 (0.13–1.23)	0.110
*Married*	0.55 (0.47–1.50)	0.553	**0.40 (0.21**–**0.78)**	**0.007**
*Widow*	0.89 (0.47–1.66)	0.707	0.60 (0.30–1.23)	0.163
*Divorced*	Ref.		Ref.	
Schooling				
*Up to 8^th^ Grade*	1.53 (0.83–2.82)	0.176	1.06 (0.45–2.51)	0.887
*Grade 9^th^ to 12^th^*	1.17 (0.85–1.62)	0.342	0.70 (0.44–1.11)	0.130
*Higher*	Ref.		Ref.	
Smoking exposure				
*Never*	0.91 (0.66–1.26)	0.583	0.90 (0.58–1.39)	0.902
*Current*	1.29 (0.74–2.25)	0.363	0.97 (0.43–2.18)	0.964
*Former*	Ref.		Ref.	
Alcohol exposure				
*None or < 1 per week*	**1.71 (1.21**–**2.42)**	**0.002**	1.15 (0.74–1.79)	0.542
*At least 1 per week*	Ref.		Ref.	
Diabetes at 1^st^ year				
*No*	0.95 (0.60–1.53)	0.842	0.72 (0.40–1.31)	
*Yes*	Ref.		Ref.	0.283
PD	1.10 (0.91–1.33)	0.344	1.13 (0.87–1.45)	0.368
At least 2 sites with PD ≥ 5 mm				
*No*	0.85 (0.62–1.15)	0.292	1.00 (0.66–1.52)	0.992
*Yes*	Ref.		Ref.	
CAL	1.01 (0.91–1.13)	0.835	**1.17 (1.03**–**1.33)**	**0.017**
No. of teeth present	1.01 (0.98–1.02)	0.675	**0.97 (0.95**–**0.99)**	**0.039**
FTU molar	0.99 (0.90–1.10)	0.905	0.87 (0.75–1.00)	0.054
FTU anterior	0.96 (0.90–1.03)	0.219	0.93 (0.85–1.01)	0.090
FTU premolar	0.98 (0.89–1.08)	0.668	0.90 (0.79–1.02)	0.104
FTU posterior	0.99 (0.94–1.05)	0.762	0.93 (0.86–1.00)	0.054
FTU total	0.99 (0.96–1.02)	0.426	**0.96 (0.92**–**0.99)**	**0.043**
Posterior FTU score	0.99 (0.96–1.03)	0.807	**0.95 (0.90**–**0.99)**	**0.049**
Total FTU score	0.99 (0.97–1.02)	0.514	**0.96 (0.93**–**0.99)**	**0.038**

*Note*: Bold estimates mean statistically significant association (p < 0.05). Posterior FTU score: represented by FTUs posterior to canines (except third molars); Total FTU score: represented by all teeth in occlusion (except third molars). In both variables, pairs of molar teeth scored 2 units and pairs of anterior teeth and premolars scored 1 unit each.

Abbreviations: CAL, clinical attachment level; FTU, functional tooth unit; OR, odds ratio; PD, probing depth; 95% CI, 95% confidence interval.

The multivariable analysis for weight alteration (Table 3) included the variables related to the oral examination, adjusted for race, marital status, education, and alcohol exposure. For weight loss, there was no significant association with any variable (*p* > 0.05). The results of the multivariable analysis indicated that for every 1 mm increase in mean CAL (OR: 1.19; 95%CI: 1.03–1.37), there is an 19% higher chance of weight gain. Moreover, for each additional tooth (OR: 0.97; 95%CI: 0.94–0.99), there is a 3% decrease in the chance of weight gain. Additionally, molar, posterior, and total FTUs, as well as posterior FTU and total FTU scores, were independently associated with weight gain. Specifically, having an occlusal pair of molars was associated with a 17% (OR: 0.83; 95%CI: 0.70–0.98) decrease in the chance of weight gain, and similar reductions of 8%, 5%, 6%, and 4% were observed for posterior FTUs (OR: 0.92; 95%CI: 0.84–0.99), total FTUs (OR: 0.95; 95%CI: 0.91–0.99), posterior FTU score (OR: 0.94; 95%CI: 0.89–0.99), and total FTU score (OR: 0.96; 95%CI: 0.92–0.99). Given that molar FTUs are included within posterior FTUs, which themselves constitute a component of total FTUs, the findings demonstrated that both posterior and total FTU measures were associated with reduced odds of weight gain. These results underscore the critical role of posterior occlusal pairs, particularly molars, in preserving masticatory capacity.

**TABLE 3:** Multivariable analyses for weight alteration (multinominal regression analyses)

Parameter	Weight loss OR (95% CI)	*p*‐Value	Weight gain OR (95% CI)	*p*‐value
PD	1.00 (0.80–1.25)	0.992	1.04 (0.78–1.38)	0.782
At least 2 sites with PD ≥ 5 mm	0.91 (0.65–1.27)	0.563	1.09 (0.69–1.72)	0.706
*No*	Ref.		Ref.	
*Yes*				
CAL	0.96 (0.85–1.08)	0.514	**1.19 (1.03**–**1.37)**	**0.016**
No. of teeth present	1.01 (0.98–1.03)	0.677	**0.97 (0.94**–**0.99)**	**0.039**
FTU molar	1.03 (0.91–1.16)	0.634	**0.83 (0.70**–**0.98)**	**0.027**
FTU anterior	0.98 (0.91–1.05)	0.578	0.93 (0.85–1.02)	0.125
FTU premolar	1.01 (0.91–1.13)	0.817	0.89 (0.77–1.03)	0.126
FTU posterior	1.01 (0.95–1.08)	0.700	**0.92 (0.84**–**0.99)**	**0.041**
FTU total	1.00 (0.96–1.04)	0.953	**0.95 (0.91**–**0.99)**	**0.043**
Posterior FTU score	1.01 (0.97–1.05)	0.669	**0.94 (0.89**–**0.99)**	**0.031**
Total FTU score	1.00 (0.97–1.03)	0.941	**0.96 (0.92**–**0.99)**	**0.031**

*Note*: All models were adjusted to sex, race, marital status, level of education, smoking exposure, alcohol exposure, diabetes, and age at 6^th^ year. Bold estimates mean statistically significant association (*p* < 0.05). Posterior FTU score: represented by FTUs posterior to canines (except third molars); Total FTU score: represented by all teeth in occlusion (except third molars). In both variables, pairs of molar teeth scored 2 units and pairs of anterior teeth and premolars scored 1 unit each.

Abbreviations: CAL, clinical attachment level; FTU, functional tooth unit; OR, odds ratio; PD, probing depth; 95% CI, 95% confidence interval.

## 4 DISCUSSION

This study evaluated the impact of FTUs and periodontal status on weight changes (loss and gain) among older adults in the health ABC study. Significant associations were found between CAL, the number of present teeth, and FTUs, especially those involving molars, with weight gain. No significant association with weight loss was identified.

Several studies have evaluated associations between weight gain and tooth loss,^13^, ^24^, ^25^ but studies evaluating weight loss are less common^26^ Studying these associations is fundamental since teeth can interfere with general health. The fact that oral health status, especially the capability of chewing food, led to an increased chance of weight gain in a well‐designed prospective cohort such as Health ABC, reinforces the importance of oral health for healthy aging. The current disconnect between oral and general health in older adults results in a lack of oral healthcare as part of healthcare policies directed to older adults.^27^ This is the case in the United States, where dental care is only covered as part of Medicare as part of the treatment of other medical conditions. The presence or absence of teeth influences individuals' food choices, and deficiencies in specific nutrient intake can precipitate the emergence of different disorders and diseases.^28^, ^29^

Unintentional weight loss is common in older adults and is associated with increased disability and mortality.^30^ In this age group, weight loss is often driven by underlying diseases such as cancer or cardiovascular conditions,^31^ systemic catabolism, or medication use, which may overshadow the role of oral health. In our study, the absence of associations between oral health measures (number of present teeth or FTUs) and weight loss may therefore be explained by the predominance of systemic factors. Moreover, many participants with tooth loss may have been rehabilitated with prostheses or implants, reducing the functional impact of oral conditions on chewing efficiency and subsequent weight reduction. Previous studies have shown that prosthetic rehabilitation can attenuate the risk of weight loss, whereas edentulism without rehabilitation remains a strong predictor.^26^, ^32^

Conversely, periodontal disease progression—particularly increased CAL—can result in tooth mobility, gingival recession, and pain during mastication.^33^ These functional limitations often lead to dietary changes, favoring softer and more processed foods rich in fats and refined carbohydrates but poor in fiber and essential nutrients.^14^ Over time, this pattern may promote higher caloric intake and weight gain, which is consistent with the association observed in our study between CAL and increased body weight.

It is also important to emphasize that weight loss in older individuals often reflects underlying morbidity and may serve as a marker of disease severity. Metalidis et al.^34^, for example, reported gastrointestinal and psychiatric disorders as common causes of weight loss, with 22% of cases linked to malignant neoplasms. Cancer, in particular, induces profound metabolic alterations, including competition for nutrients and abnormal carbohydrate, protein, and lipid metabolism, which accelerate basal metabolic rate and increase energy expenditure.^35^ In this context, we adopted the threshold of ≥5% weight change as clinically meaningful, since it reliably predicts adverse outcomes, including mortality, in older populations.^20^

The number of FTUs is often used to assess chewing efficiency since it is linked to the number of pairs of antagonist teeth.^36^ Its efficiency is influenced by the size of the tooth´s occlusal table, with molars providing the largest effective area and the greatest biting force.^37^, ^38^, ^39^ In this context, molar FTUs are included in posterior FTUs, and posterior FTUs are a component of total FTUs. The scoring system further differentiates their functional relevance by assigning greater weight to molars (2 units) compared to premolars and anterior teeth (1 unit). This distinction reflects the greater contribution of molars to chewing efficiency. The lower OR found between posterior and total FTU scores and weight gain in the present study suggests that the loss of posterior occlusal pairs, particularly molars, plays a significant role in dietary adaptations that can lead to increased caloric intake and subsequent weight gain.

In line with periodontal disease progression, as the number of teeth decreases, chewing capacity also tends to decrease.^40^ This affects dietary choices, often leading to a preference for easily chewable but less nutritious foods and a decrease in the intake of vegetables, fruits, and dietary fiber.^13^, ^25^, ^41^ The absence of molars is especially detrimental, resulting in larger food particle sizes and more chewing movements before swallowing.^42^

Consistent with this evidence, our results demonstrated that each additional tooth was associated with lower odds of weight gain (OR: 0.97), and each additional occlusal pair of molars also showed lower odds of weight gain (OR: 0.83). In addition, these findings highlight the central role of posterior teeth and periodontal health in preserving masticatory function, supporting healthier food choices, and preventing excessive weight gain.

Some authors hypothesize that obesity could be associated with inflammatory clinical periodontal parameters^43^ and increases the risk of periodontitis and caries, the main causes of tooth loss.^44^, ^45^ In contrast, others suggest that due to the masticatory insufficiency caused by tooth loss, subsequent alternative food choices can lead to obesity.^46^ It can be noted that there is still some uncertainty regarding the direction of the relationship between tooth loss and weight gain. A systematic review indicates that 75% of studies investigated tooth loss as the factor leading to weight gain or obesity.^13^ The present study aligns with the majority of literature. Despite monitoring only the weight variable over time, measured in years 2 and 6, it may be inferred that tooth loss, assessed solely in year 2, served as the initial condition.

Finally, some limitations of this study should be acknowledged. The study population included only community‐dwelling older adults who were able to respond to mailed invitations and who lived within a 2‐hour drive of the clinical centers. This may have led to underrepresentation of frailer individuals and those with lower socioeconomic status, groups that usually present with poorer oral health, higher tooth loss, and potentially different patterns of weight changes. Although representativeness is an important characteristic in epidemiological studies, the main objective of this study was to investigate potential causal associations between oral health and weight change. In this context, internal validity is more relevant than external validity, and the prospective design, standardized clinical assessments, and adjustment for multiple covariates reinforce our findings. In addition, some relevant confounders were not available in the dataset or not included in data analysis. Important determinants such as socioeconomic status, which can influence both oral health and weight change, could not be fully accounted for. Therefore, residual confounding from unmeasured variables may affect internal validity. This limitation is inherent to observational studies and should be considered when interpreting our results.

Another limitation to consider is that the periodontal examination was performed at a single point in time, making it impossible to analyze whether periodontal disease progression or incident tooth loss might have changed the results. This limits the interpretation of the findings, as it is not possible to determine whether improvements in periodontal condition or prosthetic rehabilitation during follow‐up might have mitigated weight gain or whether deterioration in oral health could have contributed to further weight changes.

A strength of the study is that a full‐mouth periodontal examination was performed, which provides a comprehensive overview of the periodontal status of the individuals evaluated. However, an accurate diagnosis of the individuals' periodontal status was not obtained because complete periodontal examination data were not available in the dataset, and information on prosthetic rehabilitation only concerned the use or non‐use of a prosthesis (in addition to the high number of missing data) and not the quality and effectiveness of the rehabilitation of missing teeth. Furthermore, even when this variable was available, a high number of missing data was detected (94.2%, data not shown). For this reason, this rehabilitation variable was not included in the present study.

## 5 CONCLUSIONS

Within the limits of this study, it can be concluded that periodontal status and loss of functional tooth units, especially molars, were associated with higher odds of weight gain among older adults over 4 years. The presence of an additional occlusal molar pair was associated with lower odds of weight gain. In contrast, oral health status was not associated with weight loss.

## AUTHOR CONTRIBUTIONS

All authors have made substantial contributions to conception and design of the study. EMS was involved in project development and data collection; JBH, FNH, and FWMGM were involved in data analysis; NMP and RPP were involved in data interpretation and description of results. MSM was engaged in writing the manuscript. All authors reviewed the manuscript critically and gave final approval of the version to be published.

## CONFLICT OF INTEREST STATEMENT

The authors declare no conflict of interest.

## Supporting information



Supporting Information

## References

[1] Cao X , Yang G , Li X , et al. Weight change across adulthood and accelerated biological aging in middle‐aged and older adults. Am J Clin Nutr. 2023;117(1):1‐11. doi:10.1016/j.ajcnut.2022.10.020 36789928 10.1016/j.ajcnut.2022.10.020

[2] Seidell JC , Visscher TL . Body weight and weight change and their health implications for the elderly. Eur J Clin Nutr. 2000;54(Suppl 3):S33‐S39. doi:10.1038/sj.ejcn.1601023 11041073 10.1038/sj.ejcn.1601023

[3] World Health Organization (WHO) . Obesity: preventing and managing the global epidemic. Report of a WHO consultation. World Health Organ Tech Rep Ser. 2000;894:1‐253. 11234459

[4] Apovian CM . Obesity: definition, comorbidities, causes, and burden. Am J Manag Care. 2016;22(3):176‐185. 27356115

[5] Engin A . The definition and prevalence of obesity and metabolic syndrome. Adv Exp Med Biol. 2017;960:1‐17. doi:10.1007/978‐3‐319‐48382‐5_1 28585193 10.1007/978-3-319-48382-5_1

[6] Veyrune JL , Miller CC , Czernichow S , et al. Impact of morbid obesity on chewing ability. Obes Surg. 2008; 18(11):1467‐1472. doi:10.1007/s11695‐008‐9443‐9 18368460 10.1007/s11695-008-9443-9

[7] Al‐Sultani HF , Field JC , Thomason JM , Moynihan PJ , The impact of replacement conventional dentures on eating experience. JDR Clin Trans Res. 2019;4(1):29‐40. doi:10.1177/2380084418803091 30931758 10.1177/2380084418803091

[8] Uy SN , Deng K , Fok CTC , Fok MR , Pelekos G , Tonetti MS . Food intake, masticatory function, tooth mobility, loss of posterior support, and diminished quality of life are associated with more advanced periodontitis stage diagnosis. J Clin Periodontol. 2022;49(3):240‐250. doi:10.1111/jcpe.13588 34935175 10.1111/jcpe.13588

[9] Hilgert JB , Hugo FN , de Sousa Mda L , Bozzetti MC . Oral status and its association with obesity in Southern Brazilian older people. Gerodontology. 2009;26(1):46‐52. doi:10.1111/j.1741‐2358.2008.00226.x 18371171 10.1111/j.1741-2358.2008.00226.x

[10] Weyant RJ , Newman AB , Kritchevsky SB , et al. Periodontal disease and weight loss in older adults. J Am Geriatr Soc. 2004;52(4):547‐553. doi:10.1111/j.1532‐5415.2004.52160.x 15066069 10.1111/j.1532-5415.2004.52160.x

[11] Ueno M , Yanagisawa T , Shinada K , et al. Masticatory ability and functional tooth units in Japanese adults. J Oral Rehabil. 2008;35(5):337‐344. doi:10.1111/j.1365‐2842.2008.01847.x 18405269 10.1111/j.1365-2842.2008.01847.x

[12] Hildebrandt GH , Dominguez BL , Schork MA , Loesche WJ . Functional units, chewing, swallowing, and food avoidance among the elderly. J Prosthet Dent. 1997;77(6):588‐595. doi:10.1016/s0022‐3913(97)70100‐8 9185051 10.1016/s0022-3913(97)70100-8

[13] Nascimento GG , Leite FR , Do LG , et al. Is there a relationship between obesity and tooth loss and edentulism? A systematic review and meta‐analysis. Obes Rev. 2016;17(7):587‐598. doi:10.1111/obr.12418 27125768 10.1111/obr.12418

[14] Hutton B , Feine J , Morais J . Is there an association between edentulism and nutritional state? J Can Dent Assoc. 2002;68(2):82‐87. 11911815

[15] Middelbeek L , Breda J . Obesity and sedentarism: reviewing the current situation within the WHO European region. Curr Obes Rep. 2013;2(1):42‐49. doi:10.1007/s13679‐013‐0054‐y

[16] Konishi M , Verdonschot RG , Kakimoto N . An investigation of tooth loss factors in elderly patients using panoramic radiographs. Oral Radiol. 2020;37(4):436‐442. doi:10.1007/s11282‐020‐00475‐6 32809096 10.1007/s11282-020-00475-6

[17] Alharbi TA , Paudel S , Gasevic D , et al. The association of weight change and all‐cause mortality in older adults: A systematic review and meta‐analysis. Age Ageing. 2021;50:697‐704. doi:10.1093/ageing/afaa231 33161429 10.1093/ageing/afaa231

[18] Von Elm E , Altman DG , Egger M , et al. STROBE Initiative. The strengthening the reporting of observational studies in Epidemiology (STROBE) statement: Guidelines for reporting observational studies. Lancet. 2007;370(9596):1453‐1457. doi:10.1016/S0140‐6736(07)61602‐X 18064739 10.1016/S0140-6736(07)61602-X

[19] Reife CM . Involuntary weight loss. Med Clin North Am. 1995;79(2):299‐313. doi:10.1016/s0025‐7125(16)30069‐4 7877392 10.1016/s0025-7125(16)30069-4

[20] Newman AB , Yanez D , Harris T , et al. Cardiovascular Study Research Group. Weight change in old age and its association with mortality. J Am Geriatr Soc. 2001;49(10):1309‐1318. doi:10.1046/j.1532‐5415.2001.49258.x 11890489 10.1046/j.1532-5415.2001.49258.x

[21] Hussain M , Ryan J , et al. Association of Weight Change With All‐Cause and Cause‐Specific Mortality Among Healthy Older Adults. JAMA Network Open. 2023;6(4):e237519. doi:10.1001/jamanetworkopen.2023.7519 10.1001/jamanetworkopen.2023.7482 PMC10087052 37036703

[22] Melo N , Ferreira AI , Silva C , et al. Influence of weight variation on long‐term mortality of patients with heart failure. Arch Cardiovasc Dis. 2023;116(8‐9):403‐410. 37574401 10.1016/j.acvd.2023.06.007

[23] Holtfreter B , Kuhr K , Borof K , et al. ACES: A new framework for the application of the 2018 periodontal status classification scheme to epidemiological survey data. J Clin Periodontol. 2024;51(5):512‐521. doi:10.1111/jcpe.13965 38385950 10.1111/jcpe.13965

[24] Singh A , Peres MA , Peres KG , et al. Gender differences in the association between tooth loss and obesity among older adults in Brazil. Rev Saude Publica. 2015;49:44. doi:10.1590/S0034‐8910.2015049005590 26270016 10.1590/S0034-8910.2015049005590 PMC4544688

[25] De Marchi RJ , Hugo FN , Hilgert JB , Padilha DM . Association between number of teeth, edentulism, and use of dentures with percentage body fat in south Brazilian community‐dwelling older people. Gerodontology. 2012;29(2):e69‐e76 . doi:10.1111/j.1741‐2358.2010.00411.x 21054508 10.1111/j.1741-2358.2010.00411.x

[26] De Andrade FB , Lebrão ML , De Oliveira Duarte YA , Santos JL . Oral health and changes in weight and waist circumference among community‐dwelling older adults in Brazil. J Am Dent Assoc. 2014;145(8):731‐736. doi:10.14219/jada.2014.35 24982279 10.14219/jada.2014.35

[27] Poudel P , Paudel G , Acharya R , et al. Oral health and healthy ageing: A scoping review. BMC Geriatr. 2024;24(1):33. 38191307 10.1186/s12877-023-04613-7 PMC10773108

[28] Lopes ENR , Silva GR , Resende CCD , et al. Prejuízos fisiológicos causados pela perda dentária e relação dos aspectos nutricionais na Odontogeriatria. Res Soc Dev. 2021;10(1):e45810111730 . doi:10.33448/rsd‐v10i1.11730

[29] Dioguardi M , Di Gioia G , Caloro GA , et al. The association between tooth loss and Alzheimer's disease: A systematic review with meta‐analysis of case control studies. Dent J (Basel). 2019;7(2):49. doi:10.3390/dj7020049 31052367 10.3390/dj7020049 PMC6630622

[30] Alibhai SM , Greenwood C , Payette H . An approach to the management of unintentional weight loss in elderly people. CMAJ. 2005;172(6):773‐780. doi:10.1503/cmaj.1031527 15767612 10.1503/cmaj.1031527 PMC552892

[31] Hussain SM , Newman AB , Beilin LJ , et al. Associations of change in body size with all‐cause and cause‐specific mortality among healthy older adults. JAMA Netw Open. 2023;6(3):e237482 . doi:10.1001/jamanetworkopen.2023.7482 37036703 10.1001/jamanetworkopen.2023.7482 PMC10087052

[32] Kusama T , Nakazawa N , Kiuchi S , et al. Dental prosthetic treatment reduced the risk of weight loss among older adults with tooth loss. J Am Geriatr Soc. 2021;69(9):2498‐2506. doi:10.1111/jgs.17279 34081343 10.1111/jgs.17279

[33] Fan J , Caton JG . Occlusal trauma and excessive occlusal forces: narrative review, case definitions, and diagnostic considerations. J Clin Periodontol. 2018;45(Suppl 18):199‐206. doi:10.1111/jcpe.12949 10.1111/jcpe.12949 29926498

[34] Metalidis C , Knockaert DC , Bobbaers H , Vanderschueren S . Involuntary weight loss: Does a negative baseline evaluation provide adequate reassurance? Eur J Intern Med. 2008;19(5):345‐349. doi:10.1016/j.ejim.2007.09.019 18549937 10.1016/j.ejim.2007.09.019

[35] Machry RV , Susin CF , Barros RC , Dal Lado L. Desnutrição em pacientes com câncer avançado: uma revisão com abordagem para o clínico. Rev AMRIGS. 2011;55(4):296‐301.

[36] Van der Bilt A , Olthoff LW , Bosman F , Oosterhaven SP . Chewing performance before and after rehabilitation of post‐canine teeth in man. J Dent Res. 1994;73(11):1677‐1683. doi:10.1177/00220345940730110201 7983253 10.1177/00220345940730110201

[37] Yurkstas AA . The masticatory act: A review. J Prosthet Dent. 1965;15(3):248‐262. doi:10.1016/0022‐3913(65)90094‐6 14267314 10.1016/0022-3913(65)90094-6

[38] Lambrecht JR . The influence of occlusal contact area on chewing performance. J Prosthet Dent. 1965;15(3):444‐450. doi:10.1016/s0022‐3913(65)80012‐9 14277474 10.1016/s0022-3913(65)80012-9

[39] Zhao L , Monahan R . Functional assessment of the stomatognathic system. Clin Plast Surg. 2007;34(3):e1‐e9 . doi:10.1016/j.cps.2007.04.003 17692690 10.1016/j.cps.2007.04.003

[40] Azevedo MS , Correa MB , Azevedo JS , Demarco FF . Dental prosthesis use and/or need impacting the oral health‐related quality of life in Brazilian adults and elders: Results from a National Survey. J Dent. 2015;43(12):1436‐1441. doi:10.1016/j.jdent.2015.10.016 26523347 10.1016/j.jdent.2015.10.016

[41] Benguigui C , Bongard V , Ruidavets JB , et al. Evaluation of oral health related to body mass index. Oral Dis. 2012;18(7):748‐755. doi:10.1111/j.1601‐0825.2012.01940.x 22548413 10.1111/j.1601-0825.2012.01940.x

[42] Yokoyama M , Shiga H , Nakajima K , et al. Masticatory performance with one missing molar. J Oral Sci. 2023;65(3):243‐245. doi:10.2334/josnusd.23‐0212 37558434 10.2334/josnusd.23-0212

[43] Da Silva FG , Pola NM , Casarin M , Silva CFE , Muniz FWMG . Association between clinical measures of gingival inflammation and obesity in adults: Systematic review and meta‐analyses. Clin Oral Investig. 2021;25(7):4281‐4298. doi:10.1007/s00784‐021‐03961‐1 10.1007/s00784-021-03961-1 33904994

[44] Flink H , Bergdahl M , Tegelberg A , et al. Prevalence of hyposalivation in relation to general health, body mass index, and remaining teeth in different age groups of adults. Community Dent Oral Epidemiol. 2008;36(6):523‐531. doi:10.1111/j.1600‐0528.2008.00432.x 18422708 10.1111/j.1600-0528.2008.00432.x

[45] Nascimento GG , Leite FR , Conceição DA , et al. Is weight gain associated with the incidence of periodontitis? A systematic review and meta‐analysis. J Clin Periodontol. 2015;42(6):495‐505. doi:10.1111/jcpe.12417 25952821 10.1111/jcpe.12417

[46] Sheiham A , Steele JG , Marcenes W , et al. The relationship among dental status, nutrient intake, and nutritional status in older people. J Dent Res. 2001;80(2):408‐413. doi:10.1177/00220345010800020201 11332523 10.1177/00220345010800020201

