# Supplementary material for: Functional tooth unit, periodontal status, and association with weight change in older adults
Source: J Periodontol. 2026 Mar 6;97(8):1807–18. doi: 10.1002/jper.70100 (PMC13502438; doi:10.1002/jper.70100)
Supplement: Supplementary file 1 [file JPER-97-1807-s001.docx]

| **Table S1.** Frequency distribution for the older adults included and excluded in the present study, along with comparison between these individuals. Excluded participants were individuals without weight at 6^th^ year. | | | |
| --- | --- | --- | --- |
|  | **Included individuals (n=903; 79.6%)** | **Excluded participants**  **(233; 20.5%)** | **P-value** |
| **Weight (year 2)**  ***Mean±SD (median – IQ)*** | 74.78±3.02  74.00 (72.00 – 77.50) | 74.54±2.79  74.00 (72.00 – 77.00) | 0.952& |
| **Sex**  ***Male – n (%)***  ***Female – n (%)*** | 463 (51.3)  440 (48.7) | 103 (44.2)  130 (55.8) | 0.054* |
| **Age (year 2)**  ***Mean±SD (median – IQ)*** | 75.28±13.11  75.30 (65.50 – 84.05) | 75.64±15.06  74.70 (65.10 – 85.30) | 0.351& |
| **Race**  ***White – n (%)***  ***Non-white – n (%)*** | 586 (64.9)  317 (35.1) | 147 (63.1)  86 (36.9) | 0.608* |
| **Marital status**  ***Single – n (%)***  ***Married – n (%)***  ***Widow – n (%)***  ***Divorced – n (%)***  ***Missing*** | 49 (5.9)  494 (59.1)  219 (26.2)  74 (8.9)  67 | 9 (4.3)  108 (51.4)  75 (35.7)  18 (8.6)  23 | 0.048* |
| **Schooling**  ***Up to 8^th^ Grade – n (%)***  ***Grade 9^th^ to 12^th^ – n (%)***  ***Higher – n (%)***  ***Missing*** | 57 (6.3)  329 (36.6)  513 (57.1)  4 | 19 (8.2)  92 (39.7)  121 (52.2)  1 | 0.334* |
| **Smoking exposure at 1^st^ year**  ***Never – n (%)***  ***Current – n (%)***  ***Former – n (%)***  ***Missing*** | 419 (46.5)  74 (8.2)  408 (45.3)  2 | 116 (49.8)  21 (9.0)  96 (41.2)  0 | 0.533* |
| **Alcohol exposure at 1^st^ year**  ***None or <1 per week – n (%)***  ***At least 1 per week – n (%)***  ***Missing*** | 606 (67.4)  293 (32.6)  4 | 154 (66.4)  78 (33.6)  1 | 0.766* |
| **Diabetes at 1^st^ year**  ***No – n (%)***  ***Yes – n (%)***  ***Missing*** | 793 (87.8)  110 (12.2)  0 | 208 (89.3)  25 (10.7)  0 | 0.541* |
| **Number of present teeth**  ***Mean±SD (median – IQ)*** | 19.82±7.25  22.00 (15.00 – 26.00) | 19.60±7.66  22.00 (15.00 – 26.00) | 0.904& |
| **PD**  ***Mean±SD (median – IQ)*** | 2.06±0.87  1.99 (1.57 – 2.51) | 2.07±0.79  1.94 (1.56 – 2.44) | 0.659& |
| **At least 2 sites with PD ≥5mm**  ***No – n (%)***  ***Yes – n (%)*** | 395 (43.7)  508 (56.3) | 102 (43.8)  131 (56.2) | 0.993* |
| **CAL**  ***Mean±SD (median – IQ)***  ***Missing*** | 2.21±1.36  1.83 (1.24 – 2.81)  2 | 2.33±1.47  1.87 (1.35 – 2.90)  1 | 0.274& |
| **FTU Molar**  ***Mean±SD (median – IQ)*** | 1.36±1.51  1.00 (0.00 – 3.00) | 1.35±1.52  1.00 (0.00 – 3.00) | 0.879& |
| **FTU Anterior**  ***Mean±SD (median – IQ)*** | 4.23±2.23  5.00 (3.00 – 6.00) | 4.20±2.34  6.00 (2.00 – 6.00) | 0.705& |
| **FTU Premolar**  ***Mean±SD (median – IQ)*** | 2.05±1.57  2.00 (0.00 – 4.00) | 2.01±1.61  2.00 (0.00 – 4.00) | 0.741& |
| **FTU posterior**  ***Mean±SD (median – IQ)*** | 3.40±2.85  3.00 (1.00 – 6.00) | 3.36±2.87  3.00 (0.00 – 6.00) | 0.743& |
| **FTU total**  ***Mean±SD (median – IQ)*** | 7.64±4.61  8.00 (4.00 – 11.00) | 7.56±4.70  8.00 (4.00 – 12.00) | 0.882& |
| **Posterior FTU score**  ***Mean±SD (median – IQ)*** | 4.76±4.27  4.00 (1.00 – 8.00) | 4.71±4.31  4.00 (0.00 – 8.00) | 0.753& |
| **Total FTU Score**  ***Mean±SD (median – IQ)*** | 8.99±5.91  9.00 (4.00 – 14.00) | 8.91±5.99  9.00 (4.00 – 14.00) | 0.898& |
| Legend: *Chi-square; &Mann-Whitney. CAL: clinical attachment level; FTU: functional tooth unit; IQ: interquartile range; PD: Probing depth; SD: standard deviation; Posterior FTU score: represented by FTUs posterior to canines (except third molars); Total FTU score: represented by all teeth in occlusion (except third molars). In both variables, pairs of molar teeth scored 2 units and pairs of anterior teeth and premolars scored 1 unit each. | | | |
